# Fructans and other water soluble carbohydrates in vegetative organs and fruits of different *Musa* spp. accessions

**DOI:** 10.3389/fpls.2015.00395

**Published:** 2015-06-09

**Authors:** Carlos I. Cruz-Cárdenas, María L. Miranda-Ham, Lizbeth A. Castro-Concha, José R. Ku-Cauich, Rudy Vergauwen, Timmy Reijnders, Wim Van den Ende, Rosa M. Escobedo-GraciaMedrano

**Affiliations:** ^1^Centro de Investigación Científica de YucatánMérida, Mexico; ^2^Laboratory of Molecular Plant Biology, KU LeuvenLeuven, Belgium

**Keywords:** banana, fructo-oligosaccharides (FOS), fruit, metabolite-phenotyping, rhizome, water soluble carbohydrates (WSC)

## Abstract

The water soluble carbohydrates (WSC) glucose, fructose, and sucrose are well-known to the great public, but fructans represent another type of WSC that deserves more attention given their prebiotic and immunomodulatory properties in the food context. Although the occurrence of inulin-type fructo-oligosaccharides (FOS) was proposed in the fruit of some banana accessions, little or no information is available neither on the exact identity of the fructan species, nor on the fructan content in different parts of banana plants and among a broader array of banana cultivars. Here, we investigated the WSC composition in leaves, pulp of ripe fruits and rhizomes from mature banana plants of 11 accessions (I to XI), including both cultivated varieties and wild *Musa* species. High performance anion exchange chromatography with integrated pulsed amperometric detection (HPAEC-IPAD) showed the presence of 1-kestotriose [GF_2_], inulobiose [F_2_], inulotriose [F_3_], 6-kestotriose and 6G-kestotriose (neokestose) fructan species in the pulp of mature fruits of different accessions, but the absence of 1,1-nystose and 1,1,1 kestopentaose and higher degree of polymerization (DP) inulin-type fructans. This fructan fingerprint points at the presence of one or more invertases that are able to use fructose and sucrose as alternative acceptor substrates. Quantification of glucose, fructose, sucrose and 1-kestotriose and principal component analysis (PCA) identified related banana groups, based on their specific WSC profiles. These data provide new insights in the biochemical diversity of wild and cultivated bananas, and shed light on potential roles that fructans may fulfill across species, during plant development and adaptation to changing environments. Furthermore, the promiscuous behavior of banana fruit invertases (sucrose and fructose as acceptor substrates besides water) provides a new avenue to boost future work on structure-function relationships on these enzymes, potentially leading to the development of genuine banana fructosyltransferases that are able to increase fructan content in banana fruits.

## Introduction

One of the most widespread alternatives to starch or sucrose as reserve carbohydrates are fructans, which are produced by about 15% of flowering plant species (Hendry, 1993). Fructans are fructose-based oligo- and polysaccharides derived from sucrose by the action of fructosyltransferases, which evolved from vacuolar invertases (VIs) (Van den Ende et al., 2011). When challenged with high sucrose, plant VIs are able to synthesize the fructan trisaccharides 1-kestotriose, 6-kestotriose and 6G-kestotriose with a typical 1-kestotriose >6G-kestotriose >6-kestotriose ratio (De Coninck et al., 2005). Fructo-oligosaccharides with a low degree of polymerization (DP) of the inulin-type [β (2,1) linkages only] are usually termed “FOS” in the context of food applications, although theoretically this term could also be used to refer to low DP fructans with other linkages (Di Bartolomeo et al., 2013). Plant inulin-type fructans are produced by the combined action of sucrose: sucrose 1-fructosyl transferase (1-SST) and fructan: fructan 1-fructosyl transferase (1-FFT) (Van den Ende, 2013). All these enzymes belong to family GH32, in which structure-function relationships have been thoroughly investigated (Van den Ende et al., 2009).

In addition to their function as reserve carbohydrates, fructans might fulfill other, perhaps more specific, roles in plant adaptation to environmental stresses (drought resistance, frost tolerance) and in osmoregulation (Asega and de Carvalho, 2004). Fructan remobilization in the stem of wheat plants under drought stress is an important contributor to grain filling (Joudi et al., 2012), but fructans also accumulate in kernels at the milky stages (Peukert et al., 2014). Antioxidant and signaling functions have been recently suggested for fructans (Van den Ende, 2013; Matros et al., 2015). Banana is also a fructan accumulator (Der Agopian et al., 2009), but the link between fructan metabolism and stress responses has not been explored in banana. Typically, biotic and abiotic stresses stand as major constraints in banana production. Interestingly, bananas are quite sensitive to drought. However, genotypes with the B genome seem to be more tolerant than those solely based on the A genome (Ravi et al., 2013).

Inulin-type fructans, like FOS, are known to confer benefits to human health by selectively promoting the growth of bifidobacteria in the colon, which has been associated with increased mineral absorption, modification of lipid metabolism, enhancement of the systemic defense function, and prevention of colon cancer and inflammatory bowel disease (Sangeetha et al., 2005). In particular, β (2,1)-fructans with low DP possess direct signaling capacity on human immune cells (Vogt et al., 2013; Peshev and Van den Ende, 2014; Di Bartolomeo and Van den Ende, 2015).

Fructan-containing plant species are found in a limited number of families, such as Liliaceae, Poaceae, and Asteraceae (Van den Ende, 2013). Despite the high fructan content in the aerial parts of some species (up to 70% DW), grasses and cereals are not used for the industrial extraction and processing of fructans. Instead, fructans can be easily extracted and processed from bulbs, tubers and tuberous roots of plants with higher harvest indexes (Sims, 2003).

The edible bananas originated from natural inter-(sub)specific- and inter-specific crosses between *M. acuminata* and *M. balbisiana*, and to a lesser extent, *M. schizocarpa* and *Australimusa* species also contributed to the gene pools of domesticated bananas (d'Hont et al., 2000). Within cultivated banana, *Musa* spp., there are four known genomes, A, B, S, and T. These correspond to the genetic constitutions of wild *Musa* species *M. acuminata* Colla (2*n* = 2*x* = 22), *M. balbisiana* Colla (2*n* = 2*x* = 22), *M. schizocarpa* N. W. Simmonds (2*n* = 2*x* = 22) and the species of section *Australimusa* (2*n* = 2*x* = 20) (d'Hont et al., 2000).

Bananas are one of the most consumed fruits worldwide and represent an important source of revenue for tropical countries, where they are also one of their main staple foods (Moshfegh et al., 1999). As such, they constitute a fundamental source of energy, vitamins and minerals for tropical countries (Wall, 2006). Different cultivars are available worldwide, with well-known agronomic characteristics and organoleptic properties, such as color, size, texture, sweetness and flavor (Aurore et al., 2009).

Although it has been reported that banana fruits contain small fructans, with significant differences in their concentrations due to cultivar identity, stage of ripening and processing (L'Homme et al., 2001; Der Agopian et al., 2009), no studies have been conducted to study their levels and types in the different organs of the plant. The aim of this study was to characterize the variation of type and content of small WSC (sucrose, glucose, and fructose) and fructans present in different vegetative organs and fruits of 11 accessions of *Musa* with different genomic constitution. Such insights may boost future work to enhance fructan content in banana plants, which could be useful for the plants' stress responses as well as to increase the nutritional properties of banana fruit.

## Materials and methods

### Plant material

In this study 11 banana accessions (I to XI) comprising eight cultivars and three fertile wild species [*M. acuminata* (A genome), *M. balbisiana* (B genome), and *M. schizocarpa* (S genome)], were utilized (Table 1). All plants were grown in the same type of soil (Cambisol, CMX) at the Uxmal Experimental Site of the Instituto Nacional de Investigaciones Forestales Agrícolas y Pecuarias (INIFAP) Yucatán, México (20° 24′ 27.72″ Lat. N, and 89° 45′ 06.66″ Long. W, elevation 44.0 meters above sea level), and tropical wet dry climate (AW0).

**Table 1:** **List of** ***Musa*** **accessions used in the present study**.

**Accession identifier**	**Species/hybrid**	***Subspecies*/subgroup**	**Putative genomic constitution**	**Accession name**	**ITC number**
I	*M. schizocarpa*	*schizocarpa*	SSw	Schizocarpa no. 1	0613
II	*M. acuminata*	*malaccensis*	AAw	Malaccensis	+
III	*M. acuminata*		AAcv	No. 110	0413
IV	*M. balbisiana*		BBw	BB-CICY	^*^
V	*Dessert bananas*	Ibota	AAA	Yangambi KM5	1123
VI	*Dessert bananas*	Cavendish	AAA	Grand Naine	^*^
VII	*Dessert bananas*	Pome	AAB	Prata Ana	0962
VIII	*Cooking bananas*	Plantain	AAB	Dominico Harton	+
IX	Dual purpose	Bluggoe	ABB	Barbaro	^**^
X	Dual purpose	Pisang Awak	ABB	Kluai Namwa Khom	0526
XI	Dual purpose	Lep Chang Kut	BBB	Lep Chang Kut	0647

w, wild species; cv, cultivated accession; ITC, International Transit Center accession number (Musa Germplasm Information System (MGIS), Bioversity International. Accessed on January 17, 2015, at http://www.crop-diversity.org/banana/). ^+^ITC under investigation,

* collected at Teapa, Tabasco, and

** *collected at Akil, Yucatan, see accession information in Valdez-Ojeda et al. (2014)*.

Leaves, fruits and rhizomes were harvested from healthy plants between 7:30 and 11:00 am during the warmest dry season from March to May 2012. The highest and the lowest temperatures over this period were 40 and 12°C, respectively with an average of 25°C. There was a 1.0 mm rain precipitation. Leaf samples were harvested from the last expanded leaf from physiologically mature plants. Leaf samples were transported to the lab in coolers, and once there, frozen with liquid nitrogen. Fruits were harvested at maturation stage 6 (Dadzie and Orchard, 1997). Ripe fruits were peeled, and the pulp was sliced and frozen in liquid nitrogen. Rhizomes were cut in halves; portions of the central cylinder were extracted and frozen in liquid nitrogen. All frozen samples were kept at −80°C until analysis. All frozen samples were lyophilized prior to extraction.

### Carbohydrate extraction, HPLC-RID and HPAEC-IPAD analyses

Carbohydrate extraction was conducted from two plants per accession with three replicates each. Lyophilized samples (0.25 g) were pulverized in liquid nitrogen and the powder extracted three times, first with 80% ethanol (two times), and finally with HPLC-grade water. Extracts were pooled and then lyophilized again. Dried samples were then resuspended in 1 mL HPLC-grade water, filtered through a 0.45 μm membrane and stored at 4°C before analysis.

Carbohydrate analysis by HPLC-RID: Filtered samples were injected into a Bio-Rad (Richmond, CA) Aminex HPX 42C (300 × 7.8 mm) HPLC column. Chromatographic analysis was performed on a liquid chromatographer Agilent 1100 (Hercules, CA, USA) equipped with a refractive index detector (HPLC-RID). Elution was obtained with HPLC-grade water at 70°C and a flow rate of 0.5 mL min^−1^. Standard curves (0 to 10 mg mL^−1^) were prepared for sucrose (Fluka®), D-glucose (Sigma-Aldrich®), D-fructose (Sigma-Aldrich®), 1-kestotriose (GF_2_; Fluka®), 1,1-nystose (GF_3_; Fluka®), and inulin from *Dahlia variabilis* (Sigma-Aldrich®).

High performance anion exchange chromatography with integrated pulsed amperometric detection (HPAEC-IPAD) was used to analyze the soluble carbohydrate composition in extracts from lyophilized samples of leaf, rhizome and fruit pulp of the three wild diploid species (*M. acuminata* ssp. *malaccensis, M. balbisiana*, and *M. schizocarpa*) (Table 1). Sample preparation and HPAEC-IPAD analysis were performed as described (Zhang et al., 2015) with D-glucose, D-fructose, sucrose, 1-kestotriose, 6-kestotriose, 6G-kestotriose, raffinose, stachyose, maltose, maltotriose, and a chicory root extract also typically containing inulobiose, inulotriose, 1,1-nystose and 1,1,1-kestopentaose (Van den Ende et al., 1996). Mild acid hydrolysis and enzymatic hydrolysis of β (2,1)-fructans with heterologous chicory 1-FEH IIa occurred as described in Vergauwen et al. (2003) and Van den Ende et al. (2011), respectively. Next to its sensitivity to 1-FEH IIa and co-elution with inulobiose from chicory, the reducing character and inulobiose nature of a peak eluting between 1-kestotriose and 6-kestotriose was further confirmed by its sensitivity to hot alkaline treatment (90 mM NaOH, pH 11, 90°C for 0, 1 and 3 h).

### Statistical analysis

A randomized complete block design (RCBD) was used with six replicates of each organ for each accession, and the data were subjected to analysis of variance (ANOVA) and Tukey's multiple comparison (SAS 9.0 institute Inc.,® Cary, NC, USA). Means evaluated were considered significantly different at *p* ≤ 0.01.

Principal component analysis (PCA) was performed for glucose, fructose, sucrose and 1-kestotriose present in the three organs (leaf, rhizomes, and fruits) of the 11 accessions (12 × 11 matrix was constructed with data). The PCA was performed by using PRIMCOMP command (SAS 9.0 institute Inc., Cary, NC, USA®).

## Results

### Analysis of type and content of water-soluble carbohydrates by HPLC-RID and HPAEC-IPAD in three organs of different Musa accessions

The WSC profiles of 11 different *Musa* accessions (Table 1) were first analyzed with HPLC-RID allowing a separation of polymerized and non-polymerized WSC (Tables 2, 3). Soluble extracts of leaves, pulp from ripe fruits and rhizomes of the different genotypes contained “putative” inulin (>DP4, peak #1, co-eluting with a *Dahlia variabilis* inulin standard), 1,1-nystose (peak #2) and 1-kestotriose (peak #3) fractions, in addition to the disaccharide sucrose (peak #4), the monosaccharides glucose (peak #5) and fructose (peak #6) and two unknown peaks. Interestingly, significant variations in the contents of these WSC were detected (Tables 2, 3).

**Table 2:** **Levels (μg g^−1^ DW) of (putative) fructan species in three organs from** ***eleven Musa*** **accessions as quantified by HPLC-RID**.

**Accession identifier**	**“Putative” Inulin (Peak # 1)**			**“Putative” 1,1-nystose (Peak # 2)**			**1-kestotriose (Peak # 3)**		
	**Leaf**	**Fruit**	**Rhizome**	**Leaf**	**Fruit**	**Rhizome**	**Leaf**	**Fruit**	**Rhizome**
I	25.34 b	20.85 d	0.00 f	0.87 d	15.20 fg	254.32 e	1.16 d	48.87 ef	0.00 f
II	13.73 e	8.09 f	4.37 d	0.97 cd	71.00 d	198.36 g	2.67 b	67.44 bcd	202.59 a
III	30.64 a	1.85 g	1.63 e	1.50 b	75.67 cd	333.11 c	0.97 d	61.61 d	0.00 f
IV	19.36 c	157.21 a	9.42 b	1.54 b	78.75 c	184.24 h	0.08 e	27.63 g	0.00 f
V	18.26 d	1.73 g	0.00 f	0.98 cd	100.60 a	233.03 f	0.00 e	74.35 ab	13.89 e
VI	11.86 d	36.25 b	6.14 c	1.02 cd	28.68 e	306.08 d	0.00 e	80.92 a	0.00 f
VII	1.13 h	33.22 b	11.49 a	1.03 cd	7.32 h	162.98 i	2.03 c	45.77 f	153.45 b
VIII	6.70 g	0.00 g	12.04 a	0.93 d	10.49 gh	427.77 a	3.27 a	53.93 e	32.69 d
IX	11.21 f	2.56 g	4.33 d	0.86 d	87.89 b	135.03 j	2.11 c	61.25 d	0.00 f
X	19.84 c	27.02 c	6.97 c	3.87 a	19.32 f	347.30 b	0.00 e	69.14 bc	124.09 c
XI	18.42 d	13.54 e	9.45 b	1.32 bc	29.17 e	225.73 f	0.00 e	64.38 cd	0.00 f

*Means sharing the same letter within a column are not significantly different (Tukey's test, P <0.001)*.

**Table 3:** **Contents of small WSC (μg g^−1^ DW) in the three organs of 11 different** ***Musa*** **accessions**.

**Accession identifier**	**Sucrose**			**Glucose**			**Fructose**		
	**Leaf**	**Fruit**	**Rhizome**	**Leaf**	**Fruit**	**Rhizome**	**Leaf**	**Fruit**	**Rhizome**
I	22.35 b	464.51 e	38.98 j	3.81 d	513.39 e	7.73 fg	3.31 b	560.51 d	3.36 h
II	16.90 d	26.30 g	65.38 f	7.51 a	13.25 k	0.00 j	2.02 cd	7.45 k	3.64 h
III	20.81 c	849.84 bc	69.47 d	4.31 cd	351.56 g	12.75 d	1.92 cd	331.44 g	9.46 f
IV	24.31 a	550.41 de	102.13 c	4.96 bc	276.98 h	75.85 b	3.00 b	165.42 i	25.31 c
V	13.33 e	825.40 bc	55.33 h	3.94 d	364.43 f	6.53 gh	1.26 de	348.28 f	11.90 e
VI	8.59 g	759.90 cd	67.58 e	2.01 e	692.93 b	3.04 i	1.59 cde	657.01 b	1.61 i
VII	9.28 g	802.99 bc	61.61 g	2.72 e	557.03 d	5.48 h	3.63 b	513.46 e	4.36 h
VIII	6.45 h	347.91 ef	69.48 d	2.23 e	575.63 c	11.19 de	15.58 a	577.05 c	15.58 d
IX	11.68 f	112.09 fg	44.79 i	2.58 e	932.94 a	9.49 ef	0.90 e	886.91 a	7.95 g
X	17.36 d	1205.43 a	181.23 a	7.32 a	244.77 i	63.45 c	2.15 c	254.29 h	40.32 a
XI	24.98 a	1037.81 ab	11.56 b	5.65 b	125.87 j	85.65 a	2.19 c	141.34 j	37.27 b

*Means sharing the same letter within a column are not significantly different (Tukey's test, P <0.001)*.

The leaf content of the “putative” inulin fraction (peak # 1) varied from 1.1 μg g^−1^ dry weight (DW) to 30.6 μg g^−1^ DW, depending on the studied genotype (Table 2). The highest value was registered for accession III, while the lowest was found in accession VII, about ~14-fold less than the mean content. In the pulp of ripe fruits, this fraction fluctuated even stronger, from 0 to 157.2 μg g^−1^ DW. The highest content was registered for accession IV, followed by accessions VI and VII. Overall, the rhizomes contained much less of this fraction (Table 2).

The presumed 1,1-nystose accumulated mainly in the rhizomes, with contents varying between 135.0 μg g^−1^ DW in the triploid accession IX and 427.8 μg g^−1^ DW in accession VIII. On the contrary, the content of 1-kestotriose was higher in the pulp of ripe fruits as compared to leaves and rhizomes, with the lowest content (27.6 μg g^−1^ DW) in accession IV and the highest (80.9 μg g^−1^ DW) in the triploid accession VI. Intriguingly, 1-kestotriose only accumulated in the rhizome of five of the eleven accessions, at varying concentrations (Table 2).

When the levels of the small WSC sucrose, glucose and fructose are compared, a clear genotypic variation is detected (Table 3). The lowest sucrose content (6.4 μg g^−1^ DW) was found in leaves of accession VIII and the highest (~25.0 μg g^−1^ DW) in accessions IV and XI. The lowest glucose content was detected in accession VI (2.0 μg g^−1^ DW) and the highest (7.5 μg g^−1^ DW) in accession II, whereas the lowest fructose (0.9 μg g^−1^ DW) was determined in accession IX and the highest (15.6 μg g^−1^ DW) in accession VIII. Rhizomes showed lower levels and even greater variation in the di– and monosaccharides, sucrose (11.6 to 181.2 μg g^−1^ DW), glucose (0.0 to 85.6 μg g^−1^ DW), and fructose (1.6 to 40.3 μg g^−1^ DW), respectively. In the pulp of ripe fruits, sucrose varied from 26.3 to 1205.4 μg g^−1^ DW, glucose from 13.2 to 932.9 μg g^−1^ DW, and fructose from 7.4 to 886.9 μg g^−1^ DW, depending on the accession (Table 3).

**Figure 1:**
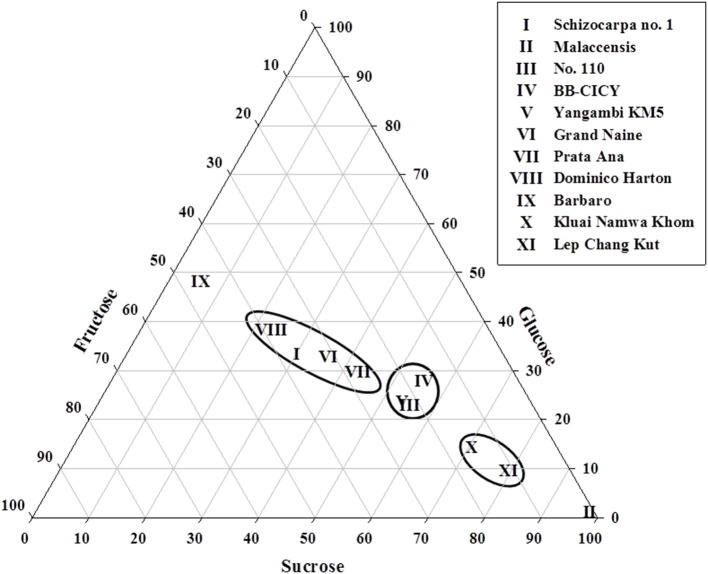
**Ternary diagram illustrating variations in sucrose, glucose and fructose contents in fruits collected from mature plants of 11** ***Musa*** **accessions**. Roman numbers of different accessions correspond to those in Table 1. Each point represents the proportional composition of carbohydrates in each individual accession. The distance from a point to the side of the triangle is proportional to the relative importance of the carbohydrate in the sample.

To examine the variations in ripe fruit of glucose, fructose, and sucrose levels among accessions in more detail, a ternary diagram was employed (Figure 1), where their location revealed the organ's pattern, given by the contribution (percentages) of each sugar. Three distinct groups can be discriminated: group 1, with four accessions (I, VI, VII, and VIII) showing low glucose (30–40%), fructose (25–40%), and sucrose (20–45%) contents; group 2, consisting of accessions III, IV, and V, with intermediate sucrose (50–60%), and low fructose (25–30%) and glucose (20–30%) concentrations. In addition, group 3 is represented by accessions X and XI showing high sucrose (>80%) and low values of glucose and fructose (10–20%). Given the WSC concentrations found in accession IX [low sucrose (~10%), intermediate fructose (40–50%) and glucose (45–55%)] and in accession II [high concentrations of sucrose (>90%) and low glucose and fructose (<10%)], they could not be included in the above-mentioned groups (Figure 1).

**Figure 2:**
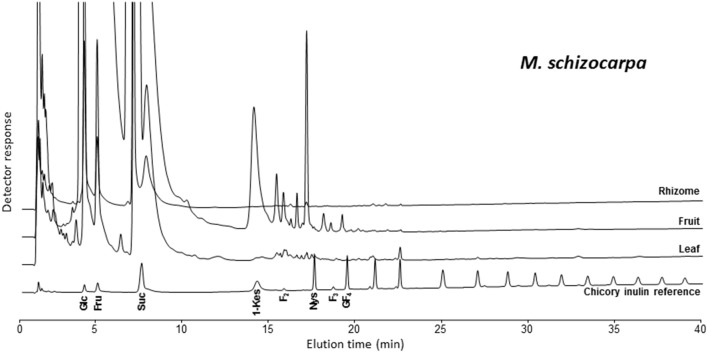
**HPAEC-IPAD saccharide patterns of leaf, fruit, and rhizome parts of** ***M. schizocarpa***. A pattern of chicory root inulin is provided alongside as a reference, containing glucose (Glc), fructose (Fru), sucrose (Suc), 1-kestotriose (1-Kes), inulobiose (F_2_), 1,1-nystose (Nys), inulotriose (F_3_), 1,1,1-kestopentaose (GF_4_) and higher molecular weight inulin-type fructans.

**Figure 3:**
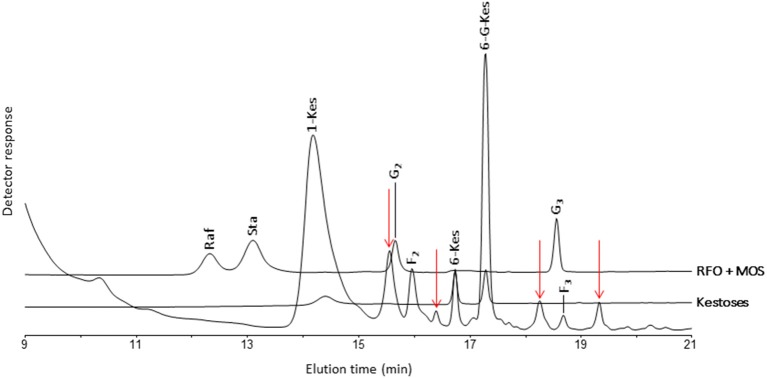
**Part of a HPAEC-IPAD chromatogram showing the saccharides in** ***M. schizocarpa*** **fruits**. Comparison with standards of 1-kestotriose (1-Kes), 6-kestotriose (6-Kes) and 6G-kestotriose (6G-Kes), raffinose (Raf), stachyose (Sta), maltose (G_2_), and maltotriose (G_3_). Besides the three kestoses, *M. schizocarpa* fruits also contain inulobiose (F_2_), inulotriose (F_3_) and four unknown saccharides that are indicated with red arrows.

The analysis of banana WSC by HPAEC-IPAD provided highly valuable qualitative information on the precise type of fructans present in pulp of mature fruit, leaf and rhizome samples of three selected wild *Musa* species. Figure 2 shows the carbohydrate patterns for *M. schizocarpa*. Very similar patterns were obtained for *M. acuminata* ssp. *malaccensis* (Figure S1) and for *M. balbisiana* (Figure S2). In addition to glucose, fructose and sucrose, a comparison with a chicory fructan profile showed that banana fruit also contained 1-kestotriose, inulobiose, and inulotriose. Surprisingly, no 1,1-nystose, 1,1,1-kestopentaose and higher DP inulin-type fructans were detected (Figure 2). Rhizomes and leaves contained lower amounts of WSC, and showed even more complex patterns (Figure 2). To unravel the identities of the peaks sequentially eluting after 1-kestotriose in banana fruit, we compared the patterns to references containing malto-oligosaccharides (MOS, maltose and maltotriose, breakdown products of starch, being very abundant in fruits), raffinose family oligosaccharides (the RFOs raffinose and stachyose) and the three kestoses: 1-kestotriose, 6-kestotriose, and 6G-kestotriose (neokestose) (Figure 3). While the banana fruit shows peaks that elute at the same retention time as the three kestoses, no MOS and RFOs could be detected in banana fruit (Figure 3). The peak eluting after 16 min, that was first thought to represent maltose, is not co-eluting with maltose (Figure S3). The presence of the three kestoses in banana fruit was further confirmed by mild acid hydrolysis (Figure S4), enzymatic hydrolysis and co-elution experiments (Figure S5). The identity of inulobiose, a major peak in banana fruit, was further confirmed by its sensitivity to hot alkaline treatment (Figure S6). The identity of 4 peaks remains unknown (see red arrows in Figure 3).

In conclusion, both non-reducing (GF_n_) and reducing inulo-n-oses or F_n_ types fructans are present in the mature fruit of the three species analyzed, *M. acuminata* ssp. *malaccensis, M. schizocarpa*, and *M. balbisiana*. The absence of any 1,1-nystose and 1,1,1 kestopentaose with HPAEC-IPAD suggests that the abundant presumed “1,1-nystose” peaks, detected with HPLC-RID, may correspond to 6G-kestotriose instead. Therefore, data of the presumptive 1,1-nystose and higher molecular weight inulins were left out from the principal component analysis (PCA).

### PCA reveals banana groups in terms of biochemical diversity in carbohydrate metabolites

**Figure 4:**
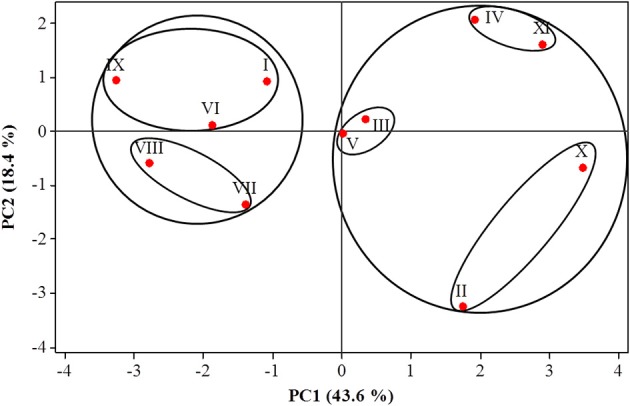
**Principal component analysis (PCA) of the four WSC, sucrose, glucose, fructose, and 1-kestotriose, present in three organs of 11** ***Musa*** **accessions**. Roman numbers of different accessions correspond to those in Table 1.

PCA analysis on the small WSC glucose, fructose, sucrose, and 1-kestotriose showed that 86.6% of the total variation in WSC within the organs and among the accessions could be explained by the first four principal components (Supplementary Table S1). Of these, the first two accounted for 62.0% of the total variation (Figure 4, PCA plot). The seven most important pairs of variables contributing to describe the first principal component PC1 were glucose and sucrose in leaves, glucose and fructose in rhizomes, and glucose, fructose and sucrose in fruit, whereas the most important variables for PC2 were 1-kestotriose and sucrose in leaves and 1-kestotriose and glucose in rhizomes. PC3 was defined by the variable 1-kestotriose in leaves and pulp from ripe fruit (Supplementary Table S1). The PCA plot, built using the Eigen-values of the two first principal components, displayed two clearly defined, major groups (Figure 4). Group 1 comprised five accessions I, VI, VII, VIII, IX and can be divided in two subgroups. The first cluster harbors accessions I, VI and IX, as defined by the PC2-positive and PC1-negative axes, whereas the second cluster contains the triploid accessions VII and VIII, as defined by both PC2 and PC1 negative axes. Group 2 enclosed a first subgroup, containing accessions V (defined near the center of the two PCs) and III (at the positive axes of PC1 and PC2). A second subgroup harbors accessions IV and XI defined by the PC1 and PC2-positive axes. Finally, a third subgroup, with accessions II and X, can be discerned (defined by positive PC1 and negative PC2 axes).

## Discussion

### Biochemical diversity of WSC in different organs from 11 accessions of banana

**Figure 5:**
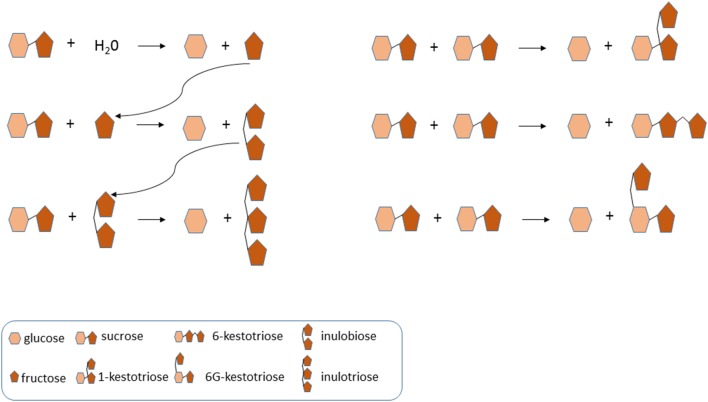
**Model of vacuolar invertase action in banana fruit, linked to the identity of FOS**. Typically, vacuolar invertases use water as acceptor substrate, producing glucose and fructose. However, increased fructose and sucrose levels may lead to the replacement of water as acceptor substrate by sucrose and fructose. In particular, inulobiose may be produced from sucrose (donor substrate) and fructose (acceptor substrate). The produced inulobiose can be used as acceptor substrate to produce inulotriose. Furthermore, the three kestoses (1-kestotriose, 6-kestotriose, and 6G-kestotriose) can be produced when sucrose acts as an acceptor substrate.

The non-reducing GF_n_ type of fructans 1-kestotriose, 6-kestotriose and 6G-kestotriose (older name, neokestose), as well as the reducing inulo-*n*-oses or F_n_ type fructans inulobiose (F_2_) and, to a lesser extent, inulotriose (F_3_), could be clearly detected in the pulp of ripe fruit, and to a lower degree, also in the leaves and rhizomes of three of the wild *Musa* accessions, which are in the ancestry of the cultivated bananas. Of the three putative inulin-type fructan peaks detected by HPLC-RID, only 1-kestotriose was validated by HPAEC-IPAD. Thus, the most important finding is that no 1,1-nystose and inulins of higher molecular weight were present in wild banana samples, demonstrating the inaccuracy of relying only on HPLC-RID data for fructan species identification. Total contents of 1-kestotriose varied not only in regard to the organ being studied, but also among the analyzed genotypes. To our knowledge, this is the first report of the presence of GF_n_ and F_n_ type of small fructans in the leaves and rhizomes of banana plants. It is noteworthy to point out that while low contents (~3.27 μg g^−1^ DW) of 1-kestotriose were found in the leaves of 63.6% of the accessions, in the pulp of ripe fruits it predominated in all accessions with intermediate values. In contrast, only 45.5% of the accessions showed important levels of 1-kestotriose in their rhizomes at concentrations that ranged between 14 to 202.6 μg g^−1^ DW. Further work needs to be done to determine the exact contents of 6-kestotriose, 6G-kestotriose, inulobiose, and inulotriose in the different organs of the cultivated accessions. In particular, little is known about the natural occurrence and physiological roles of inulo-*n*-oses in plants. Van den Ende et al. (1996) reported that under some conditions they can result from 1-FFT activity with fructan as a donor substrate and fructose as acceptor substrate. The absence of 1,1-nystose and higher DP inulin-type fructans argues against the existence of a “genuine” 1-FFT in banana. Before, the existence of a real 1-SST in banana was questioned (Der Agopian et al., 2009). These authors rather proposed the presence of a single, soluble VI with intrinsic 1-SST side activity. Plant VIs can produce the three kestoses in a certain ratio from high sucrose (De Coninck et al., 2005). Here, we also detected such typical “kestose signature” in banana fruit, suggesting that these kestoses are the product of VI activity, when subjected to strongly increased sucrose levels. Moreover, it can be speculated that one or more VI-type of enzymes can produce inulobiose from sucrose (donor substrate) and fructose (acceptor substrate), and inulotriose from sucrose (donor substrate) and inulobiose (acceptor substrate) (Figure 5). This need to be further corroborated by enzyme purification and in depth characterization of the purified enzyme (or enzymes) involved.

Previous studies presented contrasting data with regard to fructan contents in banana fruits. For instance, in banana cv. Consul premium, the only fructan detected was 1-kestotriose, with contents fluctuating between 4323 and 6020 μg g^−1^ DW, depending on the degree of maturity of the fruit (L'Homme et al., 2001). On the contrary, in eight other banana cultivars, 1-kestotriose levels were found to be much lower (297 to 1630 μg g^−1^ DW). The presence of 1,1-nystose (Der Agopian et al., 2008), 6G-kestotriose, 6-kestotriose, and bifurcose were reported in ripe fruits of the Prata cultivar (Der Agopian et al., 2009). These data contrast with our findings, pointing at lower amounts in fruits and greater variation among the analyzed genotypes. For instance, the two wild diploid accessions *M. acuminata* ssp. *malaccensis* and *M. balbisiana* exhibited differential 1-kestotriose levels (67.4 and 27.6 μg g^−1^ DW) in ripe fruits. The importance of these two species resides in their close genetic relations to present varieties of diploid and triploid bananas and plantains (d'Hont et al., 2000; Valdez-Ojeda et al., 2014).

In bananas, the physiological and biochemical roles of fructans remain to be fully elucidated, but they may function as a temporal storage or reserve of energy under adverse environmental conditions (Van den Ende, 2013). In cereals (e.g., wheat, barley, and oats), fructans accumulate temporarily in the stem and leaf sheath during vegetative growth, and are then hydrolyzed and transported as sucrose to developing grains (Peukert et al., 2014). This remobilization is thought to contribute to the final grain yield, especially when crops are subject to adverse abiotic stress (Joudi et al., 2012; Zhang et al., 2015). Additionally, fructans may be involved in hydroxyl radical scavenging (Peshev et al., 2013; Peukert et al., 2014) or in signaling events (Van den Ende, 2013). Similar functions may be proposed for fructans in banana.

Variations in the small WSC content in fruits, resulting from the relative contribution of sucrose, glucose, and fructose to the total WSC pool, support the formation of different groups (Figure 1), characterized by their phenotypic WSC profile. For instance, accessions with high levels of sucrose and low glucose and fructose contents (e.g., *M. acuminata* spp. *malaccensis*, the triploid cultivars Lep Chang Kut and Kluai Namwa Khom) formed two near clusters, whereas, an accession that was almost sucrose-free (≤10%) and contained intermediate glucose and fructose levels (e.g., Barbaro) remained separated from the other groups (Figure 1). Biochemical diversity in fruits' small WSC contents might be explained by the activity of enzymes involved in starch degradation and sucrose metabolism during fruit ripening (Fils-Lycaon et al., 2011). Both α- and β -amylases are involved in starch hydrolysis, as well as α-glucosidase and sucrose synthase functioning in a catabolic way, and their products could be converted to sucrose via sucrose phosphate synthase (SPS) and sucrose phosphate phosphatase. SPS is a key enzyme in sucrose accumulation in ripening bananas (Do Nascimento et al., 1997), and hence, a high SPS activity may contribute to direct the carbon gradient flux from starch to sucrose in banana fruits. In this regard, the activity of VIs is probably one of the key elements in the regulation of sucrose to hexose balance during banana ripening (Fils-Lycaon et al., 2011). Therefore, it would be interesting to follow more precisely the “fructan signature” and increasing fructan levels during banana fruit maturation (L'Homme et al., 2001), since fructan accumulation may in fact correlate well with increased VIs activities under (partial) starch degradation and “banana sweetening.” We propose here, as a working model for future research, that the acceptor substrate binding site of banana fruit VIs developed an affinity for sucrose and fructose (Figure 5), preventing that these sugars would reach toxic levels in the cell, and orchestrating the process of starch degradation with the ethylene-induced ripening process and with cellular respiration.

### Relationships between accessions based on the profiles of their WSC metabolites

The use of metabolite profiling analysis by PCA (Figure 4) revealed that glucose and sucrose in leaves, fructose in fruit, and sucrose, glucose, and fructose in the rhizomes were the most important traits for sorting the accessions in the first principal component that contributed to the splitting of accessions into two large groups. Furthermore, 1-kestotriose levels in leaves and rhizomes, and glucose levels in rhizomes added the most weight to the second component, for the final separation of accessions into the different groups. In summary, PCA analysis highlighted genuine differences among the three wild diploid accessions with different WSC profiles, providing some clues on the nature of these differences. Particularly interesting is the grouping of the two accessions with B genomes (*M. balbisiana* and Lep Chang Kut) from the other diploids and from triploids that share one or two A or B genomes.

Overall, the enormous variation in the 1-kestotriose contents within the 11 accessions highlights the necessity of deeper studies on the biochemistry of fructan biosynthesis within and between the different PCA subgroups, to further exploit banana biodiversity for breeding purposes, aiming to enrich fructan contents in fruits. The existence of VIs with “premature” fructosyltransferase characteristics in banana fruit is very interesting, suggesting that fructan metabolism is “emerging” in banana, which lacks “genuine” fructosyltransferases as occurring in typical fructan accumulators. Our knowledge on structure-function relationships in GH32 (Van den Ende et al., 2009) allows a fast development of “genuine” banana fructosyltransferases that may contribute to increase fructan contents in banana in the near future. Moreover, further studies are needed to elucidate the physiological roles of the different fructan types detected in the different banana organs.

### Conflict of interest statement

The authors declare that the research was conducted in the absence of any commercial or financial relationships that could be construed as a potential conflict of interest.

## Supplementary material

Figure S1 **HPAEC-IPAD saccharide patterns of leaf, fruit, and rhizome parts of *M. acuminata* ssp. *malaccensis***. A pattern of chicory root inulin is provided alongside as a reference, containing glucose (Glc), fructose (Fru), sucrose (Suc), 1-kestotriose (1-Kes), inulobiose (F_2_), 1,1-nystose (Nys), inulotriose (F_3_), 1,1,1-kestopentaose (GF_4_) and higher molecular weight inulin-type fructans.

Figure S2 **HPAEC-IPAD saccharide patterns of leaf, fruit, and rhizome parts of *M. balbisiana***. A pattern of chicory root inulin is provided alongside as a reference, containing glucose (Glc), fructose (Fru), sucrose (Suc), 1-kestotriose (1-Kes), inulobiose (F_2_), 1,1-nystose (Nys), inulotriose (F_3_), 1,1,1-kestopentaose (GF_4_) and higher molecular weight inulin-type fructans.

Figure S3 **Co-injection of banana fruit sugars with maltose**. Maltose (G_2_) does not co-elute with a peak of *M. acuminata* ssp. *malaccensis* that elutes after 16 min. 1-kestotriose (1-Kes), inulobiose (F_2_), 6-kestotriose (6-Kes), and 6G-kestotriose (6G-Kes).

Figure S4 **Mild acid hydrolysis of chicory and banana fruit sugars**. Two lower chromatograms: sucrose and inulin-type fructans from chicory (*C. intybus*) before and after mild acid hydrolysis (+ HCl). Two upper chromatograms: the three kestoses in banana fruit (*M. acuminata* ssp. *malaccensis*) before and after mild acid hydrolysis (+ HCl). Glucose (Glc), fructose (Fru), sucrose (Suc), 1-kestotriose (1-Kes), inulobiose (F_2_), 1,1-nystose (Nys), inulotriose (F_3_), 1,1,1-kestopentaose (GF_4_), 6-kestotriose (6-Kes), and 6G-kestotriose (6G-Kes).

Figure S5 **Identity of the three kestoses in banana fruit: enzymatic hydrolysis with 1-FEH and co-elution**. Treatment of the three kestoses and *M. acuminata* ssp. *malaccensis* fruit sugars with 1-FEH revealed that 1-kestotriose is an excellent substrate, while 6G-kestotriose was only slightly hydrolysed after 96 h. The identity of the kestoses in banana fruit was further confirmed by co-elution.

Figure S6 **Alkali treatment of banana fruit sugars**. Both inulobiose (F_2_) and an unknown peak (X) are sensitive to hot alkali treatment. This treatment generated massive amounts of gluconic acid, influencing the retention time and peak characteristics of 1-kestotriose (1-Kes). The levels of 1-Kes, 6-kestotriose (6-Kes) and 6G-kestotriose (6G-Kes) were not affected under this treatment.

Supplementary Table S1 **Contribution of different variables to vectors and Eigen-values in the multivariate data analysis (PCA) of WSCs in three organs from the eleven Musa accessions**.
