# Supplementary figures and images for: Fructans and other water soluble carbohydrates in vegetative organs and fruits of different Musa spp. accessions
Source: Front Plant Sci. 2015 Jun 9;6:395. doi: 10.3389/fpls.2015.00395 (PMC4460310; doi:10.3389/fpls.2015.00395)

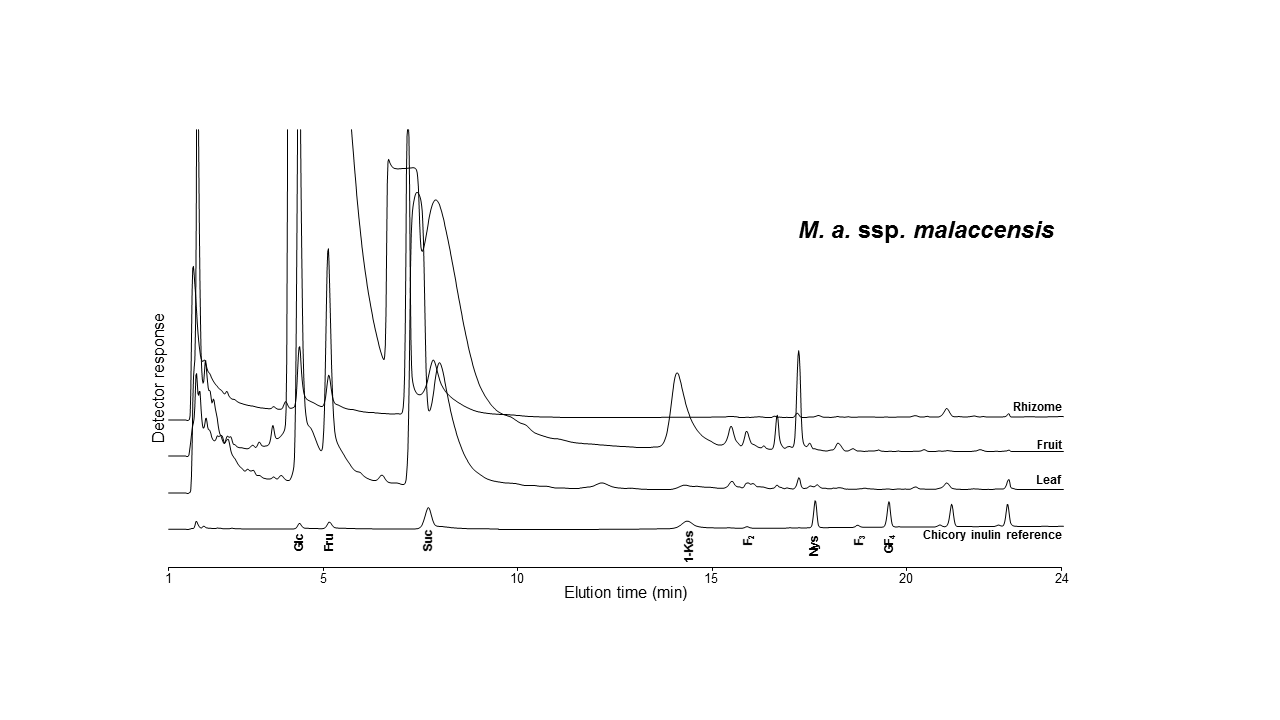

Supplement: Figure S1 — HPAEC-IPAD saccharide patterns of leaf, fruit, and rhizome parts of M. acuminata ssp. malaccensis. A pattern of chicory root inulin is provided alongside as a reference, containing glucose (Glc), fructose (Fru), sucrose (Suc), 1-kestotriose (1-Kes), inulobiose (F2), 1,1-nystose (Nys), inulotriose (F3), 1,1,1-kestopentaose (GF4) and higher molecular weight inulin-type fructans. [file Image1.TIF]

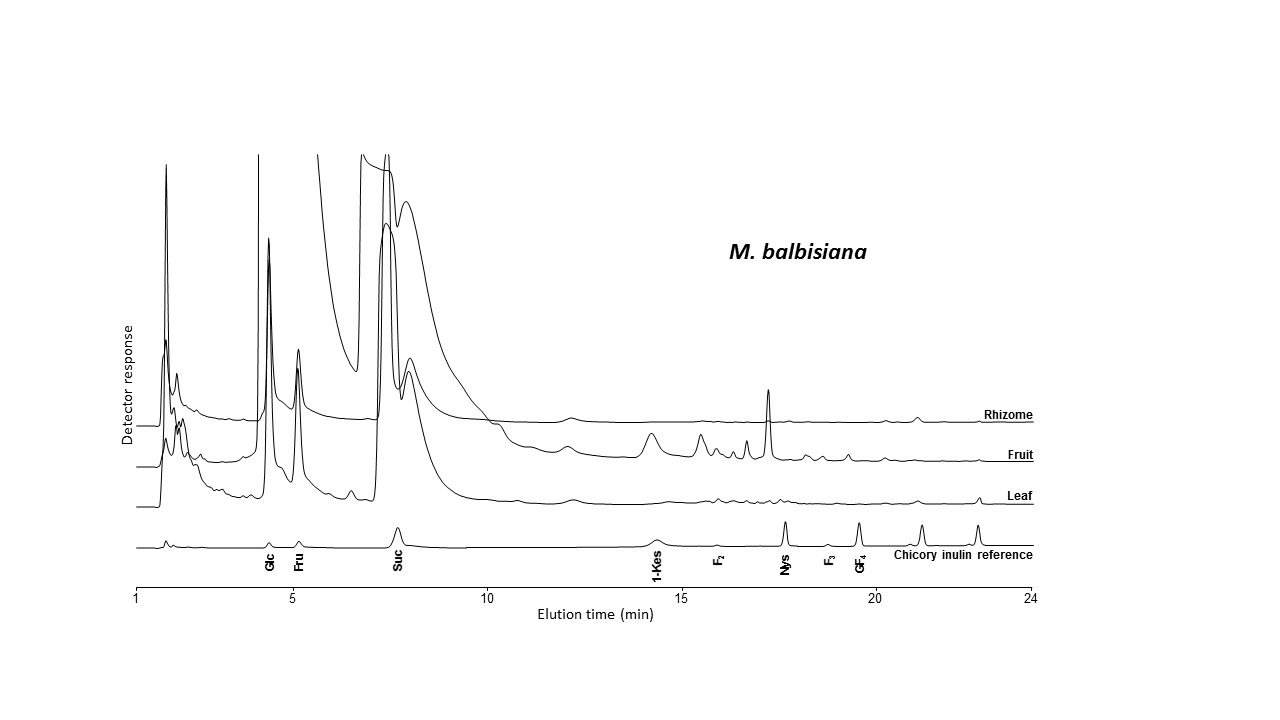

Supplement: Figure S2 — HPAEC-IPAD saccharide patterns of leaf, fruit, and rhizome parts of M. balbisiana. A pattern of chicory root inulin is provided alongside as a reference, containing glucose (Glc), fructose (Fru), sucrose (Suc), 1-kestotriose (1-Kes), inulobiose (F2), 1,1-nystose (Nys), inulotriose (F3), 1,1,1-kestopentaose (GF4) and higher molecular weight inulin-type fructans. [file Image2.TIF]

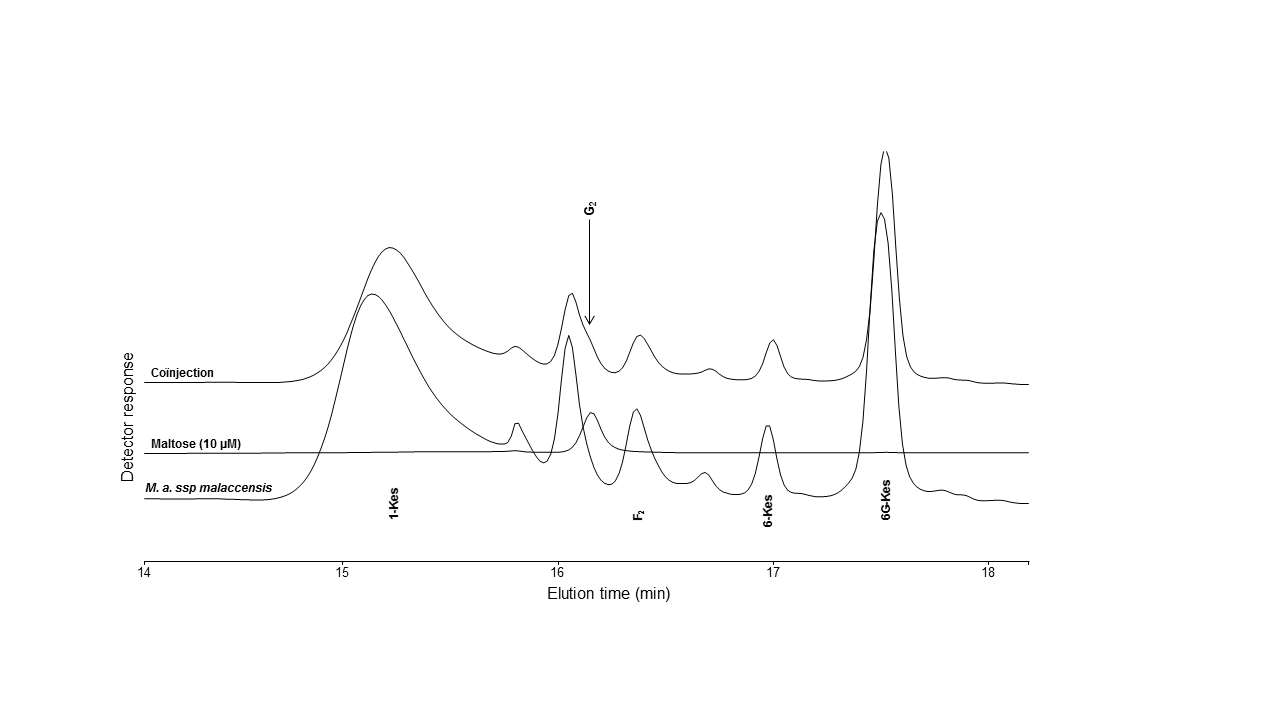

Supplement: Figure S3 — Co-injection of banana fruit sugars with maltose. Maltose (G2) does not co-elute with a peak of M. acuminata ssp. malaccensis that elutes after 16 min. 1-kestotriose (1-Kes), inulobiose (F2), 6-kestotriose (6-Kes), and 6G-kestotriose (6G-Kes). [file Image3.TIF]

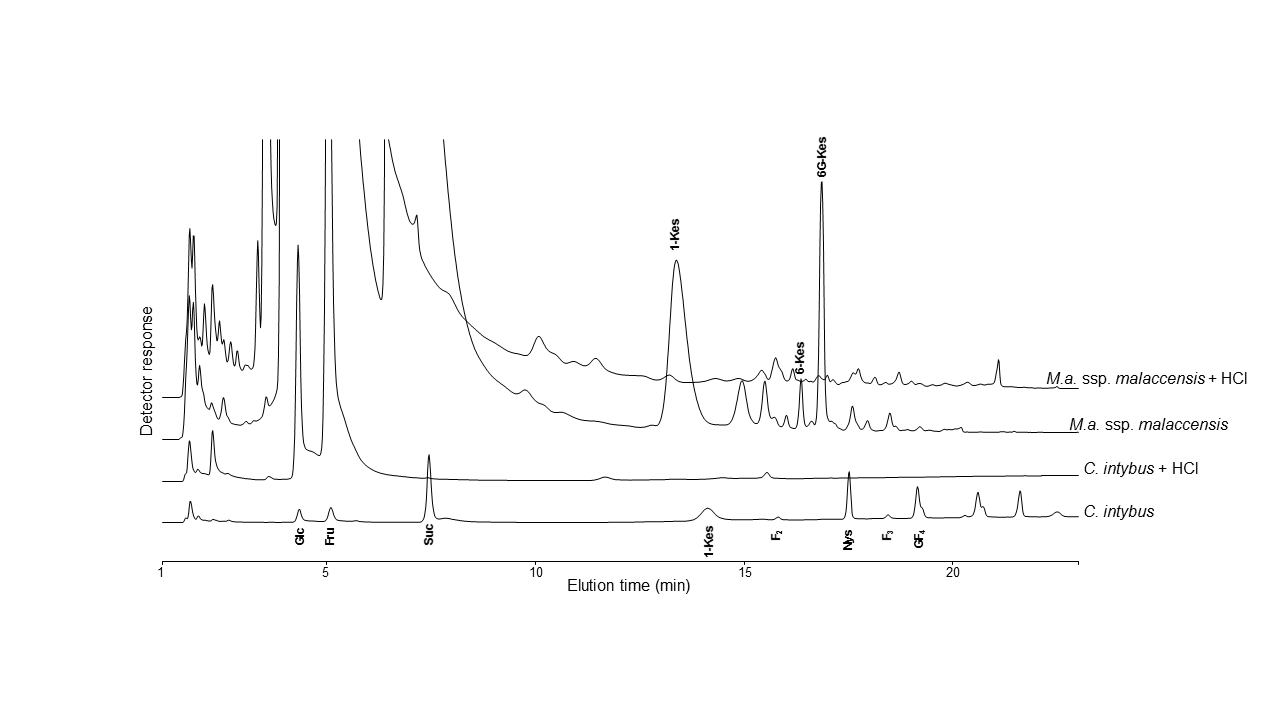

Supplement: Figure S4 — Mild acid hydrolysis of chicory and banana fruit sugars. Two lower chromatograms: sucrose and inulin-type fructans from chicory (C. intybus) before and after mild acid hydrolysis (+ HCl). Two upper chromatograms: the three kestoses in banana fruit (M. acuminata ssp. malaccensis) before and after mild acid hydrolysis (+ HCl). Glucose (Glc), fructose (Fru), sucrose (Suc), 1-kestotriose (1-Kes), inulobiose (F2), 1,1-nystose (Nys), inulotriose (F3), 1,1,1-kestopentaose (GF4), 6-kestotriose (6-Kes), and 6G-kestotriose (6G-Kes). [file Image4.TIF]

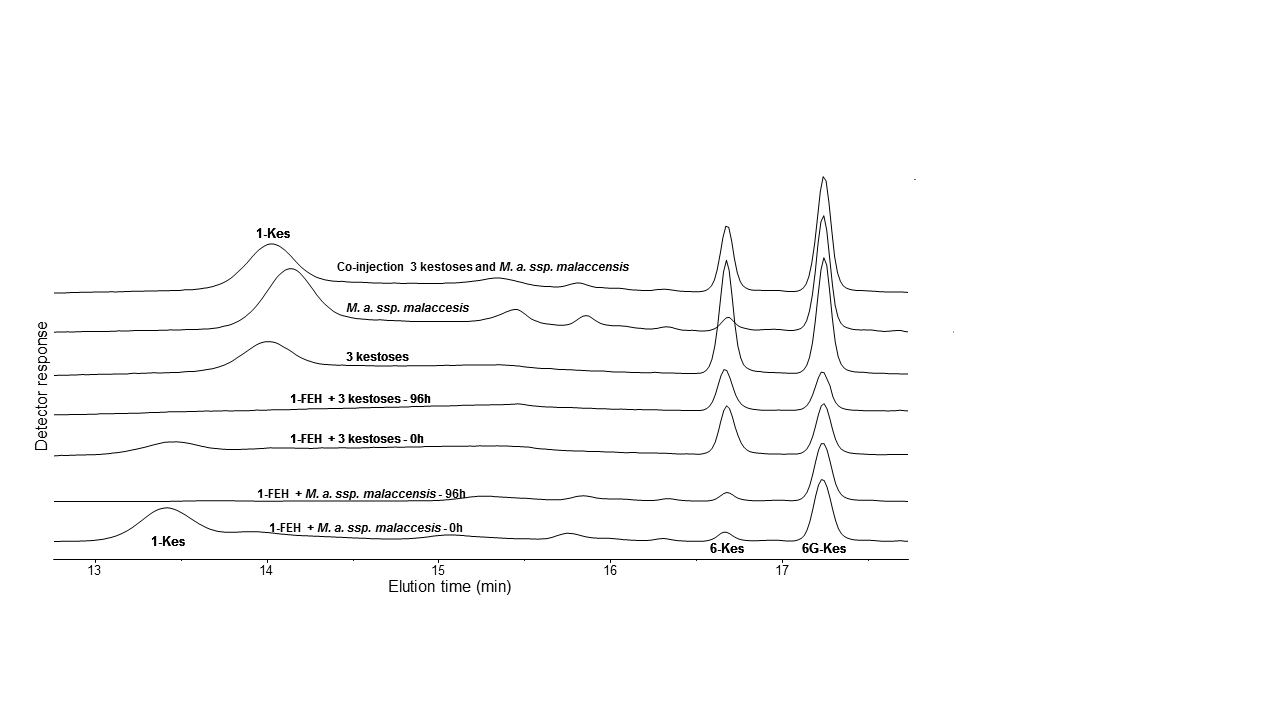

Supplement: Figure S5 — Identity of the three kestoses in banana fruit: enzymatic hydrolysis with 1-FEH and co-elution. Treatment of the three kestoses and M. acuminata ssp. malaccensis fruit sugars with 1-FEH revealed that 1-kestotriose is an excellent substrate, while 6G-kestotriose was only slightly hydrolysed after 96 h. The identity of the kestoses in banana fruit was further confirmed by co-elution. [file Image5.TIF]

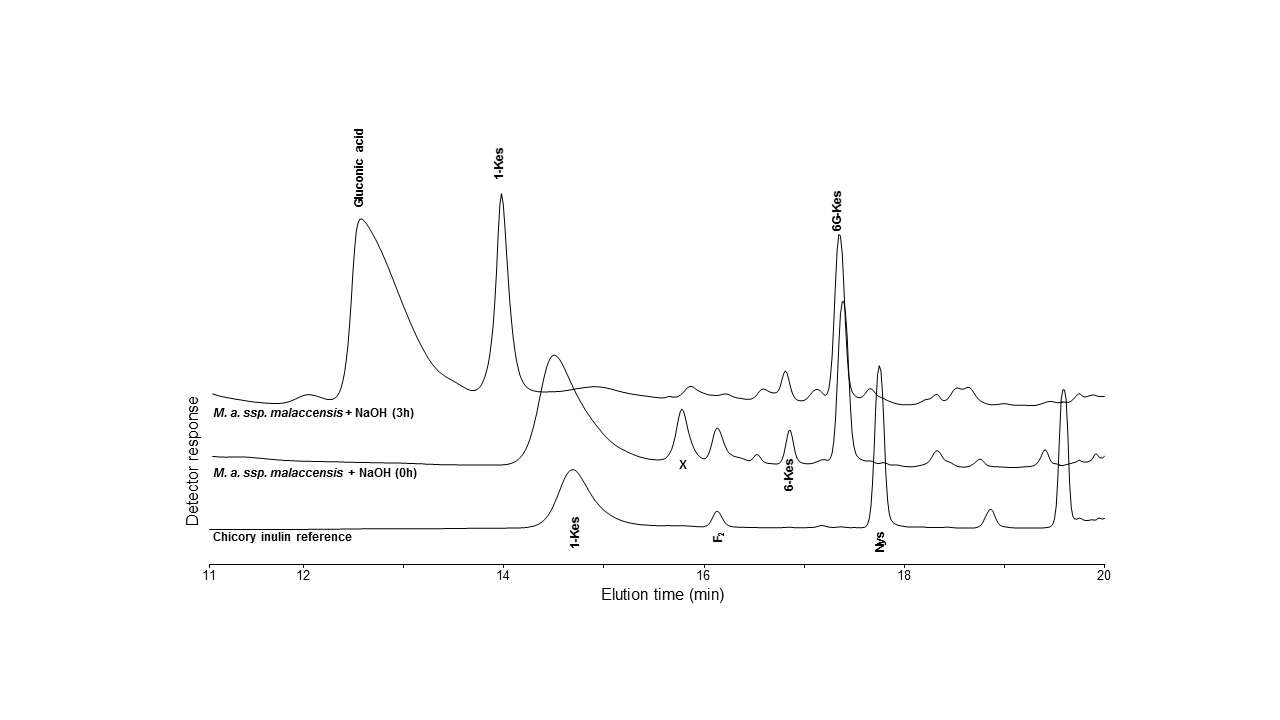

Supplement: Figure S6 — Alkali treatment of banana fruit sugars. Both inulobiose (F2) and an unknown peak (X) are sensitive to hot alkali treatment. This treatment generated massive amounts of gluconic acid, influencing the retention time and peak characteristics of 1-kestotriose (1-Kes). The levels of 1-Kes, 6-kestotriose (6-Kes) and 6G-kestotriose (6G-Kes) were not affected under this treatment. [file Image6.TIF]
