# Supplementary material for: Fructans and other water soluble carbohydrates in vegetative organs and fruits of different Musa spp. accessions
Source: Front Plant Sci. 2015 Jun 9;6:395. doi: 10.3389/fpls.2015.00395 (PMC4460310; doi:10.3389/fpls.2015.00395)
Supplement: Supplementary Table S1 — Contribution of different variables to vectors and Eigen-values in the multivariate data analysis (PCA) of WSCs in three organs from the eleven Musa accessions. [file Table1.DOCX]

**Supplementary Table.** Contribution of different variables to vectors and Eigen-values in the multivariate data analysis (PCA) of WSCs in three organs from the eleven *Musa* accessions (see Table 1 under the results section).

|  | **PRINCIPAL COMPONENTS** | | | |
| --- | --- | --- | --- | --- |
|  | **PC1** | **PC2** | **PC3** | **PC4** |
| **EIGEN-VALUES** | 5.232 | 2.204 | 1.708 | 1.273 |
| **PROPORTION (%)** | 43.6 | 18.4 | 14.2 | 10.6 |
| **CUMULATIVE (%)** | 43.6 | 62.0 | 76.2 | 86.6 |
|  | **EIGEN-VECTORS** | | | |
| **VARIABLES** | **PC 1** | **PC2** | **PC3** | **PC4** |
| **KESTOSE LEAVES** | -0.233 | -0.369 | 0.400 | -0.195 |
| **SUCROSE LEAVES** | 0.310 | 0.311 | 0.033 | -0.396 |
| **GLUCOSE LEAVES** | 0.394 | -0.186 | 0.033 | -0.076 |
| **FRUCTOSE LEAVES** | -0.158 | -0.088 | 0.565 | 0.188 |
| **KESTOSE FRUIT** | 0.002 | -0.228 | -0.576 | 0.250 |
| **SUCROSE FRUIT** | 0.349 | -0.268 | -0.232 | 0.082 |
| **GLUCOSE FRUIT** | -0.389 | 0.167 | -0.090 | 0.238 |
| **FRUCTOSE FRUIT** | -0.390 | 0.145 | -0.107 | 0.233 |
| **KESTOSE RHYSOME** | 0.123 | -0.589 | 0.123 | 0.016 |
| **SUCROSE RHIZOME** | 0.178 | -0.135 | 0.130 | 0.636 |
| **GLUCOSE RHIZOME** | 0.316 | 0.363 | 0.204 | 0.174 |
| **FRUCTOSE RHIZOME** | 0.312 | 0.235 | 0.198 | 0.386 |
